# Nasotracheal enterococcal carriage and resistomes: detection of *optrA-*, *poxtA-* and *cfrD-*carrying strains in migratory birds, livestock, pets, and in-contact humans in Spain

**DOI:** 10.1007/s10096-023-04579-9

**Published:** 2023-03-09

**Authors:** Idris Nasir Abdullahi, Carmen Lozano, Guillermo Juárez-Fernández, Ursula Höfle, Carmen Simón, Silvia Rueda, Angela Martínez, Sandra Álvarez-Martínez, Paula Eguizábal, Beatriz Martínez-Cámara, Myriam Zarazaga, Carmen Torres

**Affiliations:** 1 Area of Biochemistry and Molecular Biology, OneHealth-UR Research Group, University of La Rioja, Logroño, 26006 Spain; 2 Spanish Wildlife Research Institute IREC (CSIC-UCLM-JCCM), SaBio (Health and Biotechnology) Research Group, Ciudad Real, Spain; 3 Faculty of Veterinary Medicine, University of Zaragoza, Zaragoza, Spain

**Keywords:** Nasal enterococci, Antimicrobial resistomes, Linezolid resistance, *optrA*, *cfrD*, *poxtA*, Migratory birds, Livestock, Pets

## Abstract

This study determined the carriage rates and antimicrobial resistance (AMR) genes of enterococci from nasotracheal samples of three healthy animal species and in-contact humans. Nasal samples were collected from 27 dog-owning households (34 dogs, 41 humans) and 4 pig-farms (40 pigs, 10 pig-farmers), and they were processed for enterococci recovery (MALDI-TOF–MS identification). Also, a collection of 144 enterococci previously recovered of tracheal/nasal samples from 87 white stork nestlings were characterized. The AMR phenotypes were determined in all enterococci and AMR genes were studied by PCR/sequencing. MultiLocus-Sequence-Typing was performed for selected isolates. About 72.5% and 60% of the pigs and pig-farmers, and 29.4% and 4.9%, of healthy dogs and owners were enterococci nasal carriers, respectively. In storks, 43.5% of tracheal and 69.2% of nasal samples had enterococci carriages. Enterococci carrying multidrug-resistance phenotype was identified in 72.5%/40.0%/50.0%/23.5%/1.1% of pigs/pig-farmers/dogs/dogs’ owners/storks, respectively. Of special relevance was the detection of linezolid-resistant enterococci (LRE) in (a) 33.3% of pigs (*E. faecalis*-carrying *optrA* and/or *cfrD* of ST59, ST330 or ST474 lineages; *E. casseliflavus*-carrying *optrA* and *cfrD*); (b) 10% of pig farmers (*E. faecalis*-ST330-carrying *optrA*); (c) 2.9% of dogs (*E. faecalis-*ST585-carrying *optrA*); and (d) 1.7% of storks (*E. faecium*-ST1736-carrying *poxtA*). The *fexA* gene was found in all *optrA*-positive *E. faecalis* and *E. casseliflavus* isolates, while *fexB* was detected in the *poxtA-*positive *E. faecium* isolate. The enterococci diversity and AMR rates from the four hosts reflect differences in antimicrobial selection pressure. The detection of LRE carrying acquired and transferable genes in all the hosts emphasizes the need to monitor LRE using a One-Health approach.

**Supplementary Information:**

The online version contains supplementary material available at 10.1007/s10096-023-04579-9.

## Introduction

*Enterococcus* spp. are commensals and predominantly found in the intestinal habitat, but they might be translocated to other animal tissues or organs [1]. Among the over 50 different enterococci species, *Enterococcus faecium* (*E. faecium*) and *Enterococcus faecalis* (*E. faecalis*) constitute most of the gastrointestinal tract (GI) enterococci communities in humans [2]. However, in livestock, *E. faecium*, *E. cecorum*, *E. faecalis* and, to some extent, *E. hirae* predominate [3]. In contrast, *E. mundtii* and *E. casseliflavus* are commonly found in plant and environmental samples [2, 4]. Moreover, the ecologic-epidemiology of *E. faecalis* and *E. faecium* has shown animal food (such as pork) and the environment (sewage, soil, and water) as common colonized items [4, 5].

Enterococci are very hardy organisms; they can sustain various adverse conditions and survive for several months in the environment [2]. These attributes make enterococci challenging to control once established in a hospital, household, or pig-farm setting. Enterococci are also suitable as important key indicator bacteria for veterinary and human resistance surveillance systems [4, 6]. Enterococci can easily acquire antimicrobial resistance (AMR) through mutations or the acquisition of antimicrobial resistance genes (ARGs), included in plasmids and transposons [7].

The livestock industry plays an important role in the transmission of multidrug-resistant (MDR) enterococci isolates due to the close interaction between farmers, livestock, and the farm environment [7, 8]. However, a new European Union law now prohibits the use of antimicrobial agents as prophylactics in feeds [9]. The pervasive selection of resistant bacteria in livestock facilitates the persistence and dissemination of MDR isolates to other animals and humans [7]. The spread of such MDR isolates can occur through direct (consumption/handling of contaminated food, direct contact with farmers/veterinarians) or indirect routes (animal waste handling, contaminated groundwater or surfaces) [10].

Among antimicrobial resistance transmission, it is important to remark that the widespread use of chloramphenicol in the past for farm animals has been able to select bacteria resistant to this agent and maybe to other clinically important antibiotics (by co-selection) [11]. Linezolid is one of the most important treatment options for severe infections by enterococci, including vancomycin-resistant enterococci (VRE). Increasing reports on linezolid-resistant enterococci (LRE) detected throughout the agricultural sector including poultry, pigs and cattle indicate that linezolid resistance might be co-selected by the use of chloramphenicol in livestock, with potentially serious consequences for public health [12–14].

Interestingly, a study on farm animals found limited sharing of isolates and resistance genes between livestock and humans, except for some pig isolates that were genetically related to hospital-associated isolates [15]. By contrast, dogs may be a reservoir of hospital-associated *E. faecium* clones and may form a higher risk for zoonotic transfer to humans [16]. Occupational contact with livestock plays a major role in certain professions, such as veterinarians, slaughterhouse workers or farmers [17, 18]. However, in the case of pets, not only veterinarians but also animal owners are at risk of acquiring MDR zoonotic bacterial pathogens [19]. It has been observed as enterococci, in particular *E. faecalis* and *E. faecium*, have zoonotic potential [20]. Dogs are companion animals that have been in close contact with humans since ancient times, which increases the likelihood of the transmission of bacteria between these animals and their owners [21].

Finally, wildlife has been considered key players in the carriage and transmission of AMR as many of them, especially the migratory birds (such as storks), could be dynamic and move along distance across a variety of natural environments, landfills, and livestock farms [22]. Migratory birds occasionally come in contact with antibiotic residues in livestock carcasses or manure and they could carry and disseminate AMR bacteria such as *Enterococcus* of public health concerns [22].

Studies on LRE of animal origin are available in some European countries, including Spain. However, studies reported on nasal LRE carriage are very rare. Especially among *Enterococcus* species other than *E. faecalis* and *E. faecium*. This prospective comparative study sought to determine the prevalence of nasal carriage of *Enterococcus* sp. and the molecular characterization of isolates in a population of healthy dogs, dog-owning households, pigs and pig-farmers, and storks in Spain, with special focus on linezolid resistance characterization.

## Material and methods

### Study participants’ descriptions and samples analyses

Nasal samples were obtained during 2021–2022 from animals and humans of the following origins: (a) 27 dog-owning households (34 dogs, 41 humans) in La Rioja region (Spain); (b) 40 pigs of 4 farms (A–D) comprising 10 pigs from each farm from the Aragon region (Spain), and 10 workers of the pig-farms (2, 3, 2 and 3 humans in farms A–D, respectively). The nasal samples were obtained using sterile swabs with conservation media (Amies, City, Country), and were used for enterococci recovery as below indicated. Moreover, a collection of 144 enterococci previously recovered from nasal and tracheal samples of 87 nestling white storks [23] were included in this study for phenotypic and genotypic characterization of antimicrobial resistance and molecular typing; these nestlings corresponded to four different colonies of Ciudad Real region (Center-South of Spain) with parent storks foraging on different habitats (colonies 1 and 2: located in natural habitat; colonies 3 and 4: foraging in landfills). The storks isolates were of the following species (number of isolates): *E. faecalis* (78), *E. faecium* (44), *E. cecorum* (8), *E. casseliflavus* (5), *E. gallinarum* (2), *E. durans* (2), *E. hirae* (1) and *E. canis* (*n* = 1).

Collected nasal and tracheal samples were enriched in brain heart infusion broth (BHI; Condalab, Madrid, Spain) supplemented with 6.5% NaCl and incubated for 24 h at 37 °C. After overnight incubation, different dilutions of the broth culture were carefully dispensed onto blood agar (BioMerieux) and ChromAgar LIN (CHROMagar™ LIN, Paris, France) plates and incubated for 24 h at 37 °C, for enterococci recovery. The CHROMAgar™ medium has been previously shown to have high sensitivity and specificity on pure linezolid resistant enterococci and staphylococci isolates [24]. After overnight growth, 2 to 5 different colonies per sample with the morphology of enterococci were randomly selected and identified by matrix-assisted laser desorption/ionization time-of-flight (MALDI-TOF; Bruker Daltonics, Bremen, Germany) using the standard extraction protocol recommended by Bruker.

All sampling procedures were performed following all applicable international, national, and/or institutional guidelines for human samples experiments (as described in the revised Helsinki declaration) and for ethical use of animals, specifically directive 2010/63/EU and Spanish laws 9/2003 and 32/2007, and RD 178/2004 and RD 1201/2005. All procedures were approved by the ethical committees of the University of La Rioja, the University of Zaragoza and the University of Castilla La Mancha of Spain.

### Enterococci DNA extraction

The DNA extraction of enterococci isolates of all origins was performed using InstaGene Matrix (Bio-Rad Laboratories, Hercules, CA, USA), according to the manufacturer’s instructions. Briefly, pure and fresh isolated colonies were suspended in 1000 μL of sterile Milli-Q water, thoroughly mixed by vortex, and centrifuged at 12,000 revolutions per minute for 3 min. The supernatant was carefully eliminated and 20 μL of InstaGene matrix was added to the sediment, thoroughly mixed by vortex and incubated in a bath for 20 min at 56 °C. Later, reincubated for 8 min at 100 °C and centrifuged at 12,000 revolutions per minute for 3 min. The DNA was stored at − 20 °C.

### Antimicrobial susceptibility testing and detection of AMR genes

The antimicrobial susceptibility testing was conducted on all enterococci isolates following the recommendations and breakpoints of the European Committee on Antimicrobial Susceptibility Testing (EUCAST, 2022). The antimicrobial agents tested were as follows (µg/disk): penicillin (10), erythromycin (15), gentamicin (120), streptomycin (300), tetracycline (30), ciprofloxacin (5), chloramphenicol (30), linezolid (10), vancomycin (30) and teicoplanin (30).

Based on the antimicrobial resistance phenotypes of all enterococci, isolates from different samples or the same sample but of different species and/or different AMR phenotypes were selected for further studies (considered as distinct isolates) (Supplementary Table S1). This collection was characterized to determine the AMR genes and genetic lineages. MDR was defined by phenotypic resistance to three or more families of antibiotics. The minimum inhibition concentration (MIC) of all isolates carrying linezolid resistance genes was tested using bioMérieux Linezolid Etest® strips (Marcy l’Étoile, France), and the results were interpreted following the EUCAST 2022 breakpoint.

The corresponding AMR genes for all antibiotics were tested by PCRs and selected according to the resistance phenotype: erythromycin (*ermA*, *ermB*, *ermC*, and *ermT*), gentamicin (*aac6’-aph2″*), streptomycin (*str* and *ant6’*), tetracycline (*tetL*, *tetM*, and *tetK*), chloramphenicol (*catpC221*, *catpC223*, *catpC194*, *catA*, *fexA*, and *fexB*), linezolid (*optrA*, *poxtA*, *cfr*, *cfrB*, and *cfrD*), and vancomycin (*vanA* and *vanB*). Specifically, all chloramphenicol-resistant isolates were tested for the possible presence of linezolid resistance genes and mutations in the 23S rRNA, regardless of the linezolid zone of inhibition by antibiogram. All isolates positive for linezolid resistance genes were confirmed by sequencing.

### Genetic characterization

Multilocus sequence typing (MLST) was performed for *E. faecalis* and *E. faecium* isolates carrying linezolid resistance genes. The 7 housekeeping genes (*gdh*, *gyd*, *pstS*, *gki*, *aroE*, *xpt* and *yqiL* of *E. faecalis*; *adK*, *atpA*, *ddl*, *gdh*, *gyd*, *pstS* and *purK* of *E. faecium*) were amplified and sequenced, and Sequenced Types (ST) were assigned from analyses on the MLST database (https://pubmlst.org/organisms/enterococcus-faecalis). Primers and conditions of PCRs for the AMR genes tested and for MLST typing are included in Supplementary Table S2.

### Data management and analyses

Data collected were verified, entered and analysed with Statistical Package for Social Sciences (SPSS) Version 26 (IBM, California, USA). Data were reported as numbers and percentages (for categorical variables). Tables and charts were plotted. Data were subjected to univariate logistics to compute Odds Ratio (OR) and chi-squared test at 95% confidence interval (95%CI) between the carriage rate of enterococci and some categorical variables (such as household densities, animal species and AMR phenotypes). A significant association was set < 0.05 probability value.

## Results

### Nasal enterococcal carriage rate in healthy pigs and pig-farmers

**Fig. 1:**
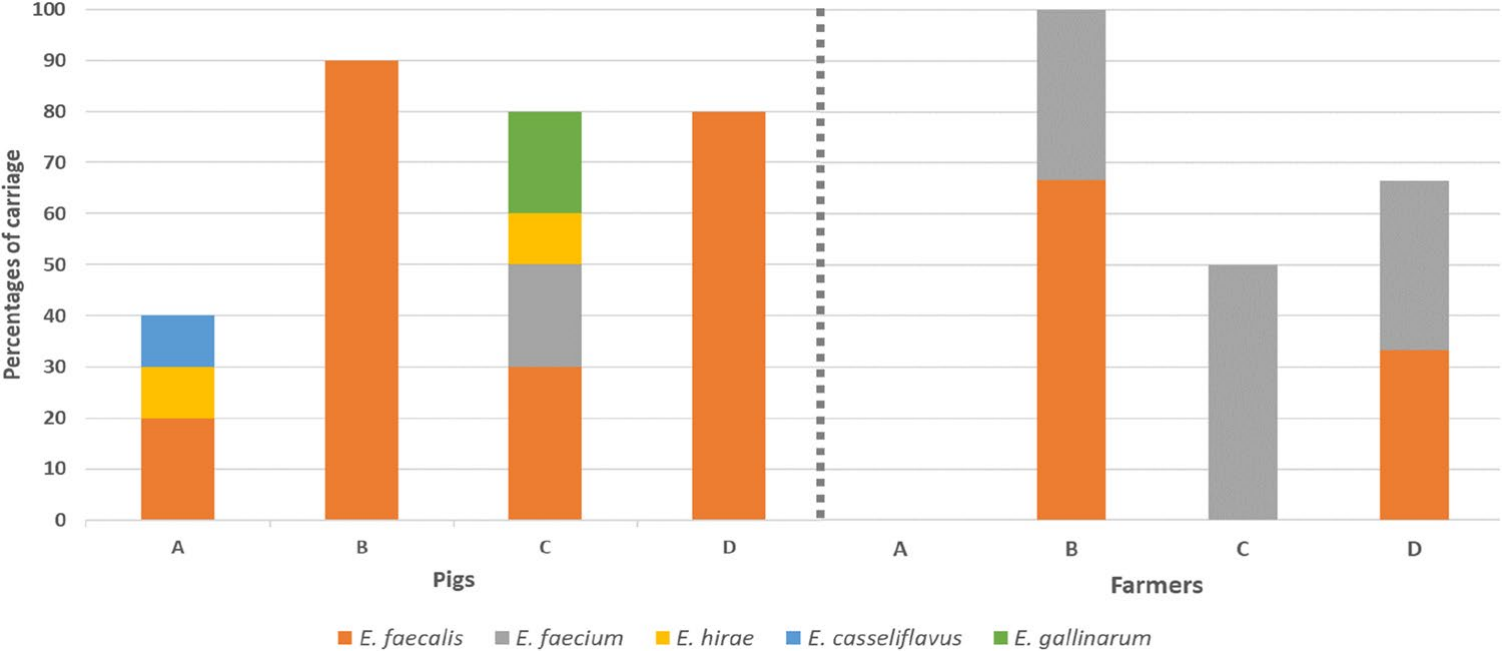
Nasal enterococci carriage detected in pigs and farmers in the four pig-farms (A, B, C and D). The number of individuals sampled from pigs and farmers were 40 and 10, respectively

Enterococci nasal carriage was found in all the farms. In total, 51 enterococci isolates (43 from pigs, and 8 from pig-farmers) were recovered. Of the pigs’ isolates, 34, 4, 2, 2, and 1 were *E. faecalis*, *E. faecium*, *E. gallinarum*, *E. hirae* and *E. casseliflavus*, respectively. However, among the enterococci isolates from the pig-farmers, they were only 4 *E. faecium* and 4 *E. faecalis* isolates (Fig. 1). Of the 40 pigs studied, 29 (72.5%) were enterococci nasal carriers. Of these, 4 (40%), 9 (90%), 8 (80%) and 8 (80%) were obtained in farms-A to D, respectively (Fig. 1). Specifically, nasal carriage of *E. faecalis* (*n* = 2), *E. casseliflavus* (*n* = 1) and *E. hirae* (*n* = 1) were identified in pigs of farm-A; *E. faecalis* (*n* = 9) from pigs of farm-B; *E. faecalis* (*n* = 3), *E. faecium* (*n* = 2), *E. hirae* (*n* = 1), and *E. gallinarum* (*n* = 2) from farm-C; and *E. faecalis* (*n* = 6), *E. faecalis/E. faecium* co-carriage (*n* = 2) from farm-D (Fig. 1). Conversely, all the three farmers (100.0%) in farm-B were enterococci nasal carriers (66.7% *E. faecalis* and 33.3% *E. faecium*); 1 of the 2 farmers (50.0%) in farm-C was a nasal carrier (*E. faecium*); 2 of the 3 farmers of farm-D (66.7%) were nasal carriers (50.0% *E. faecalis* and 50.0% *E. faecium*), but none of the farmers in farm-A were enterococci nasal carriers (Fig. 1).

### Antimicrobial resistomes of *Enterococcus* sp. isolates from pigs farms

In farms-A to -D, all the *E. faecalis* isolates from pigs and farmers were MDR (Table 1). Multiresistance was also detected in other species such as the *E. casseliflavus* (carrying *fexA*, *optrA*, *cfrD*, *tetK*, *tetL*, and *ermB* genes)*, E. hirae* and *E. gallinarum* (carrying *ermB*, *tetM*, and *ant6* genes).

**Table 1:** Intra-sample and intra-species variation of resistomes of *Enterococcu*s sp. isolates from all pigs and pig-farmers

Farm	Host/carriers	*Enterococcus *sp.	No. of isolates	AMR phenotypes^a,b^	LZD MIC (μg/ml)^c^	AMR genes detected^a^	Sequence type
Farm-A	Pig/2	*E. faecalis*	2	CLO^2^-TET^2^-ERY^2^-CIP^2^ STR^2^	10, 12	*fexA*^2^*, optrA*^2^*, tetM*^2^*, ermA*^2^*, ermB*^2^*, ant6′*^2^	ST330
	Pig/1	*E. hirae*	1	PEN^1^-TET^1^-ERY^1^-STR^1^	NT	*ermB*^1^	NT
	Pig/1	*E. casseliflavus*	1	STR^1^-TET^1^-ERY^1^-CLO^1^	8	*fexA*^1^*, cfrD*^1^*, optrA*^1^*, tetK*^1^*, tetL*^1^*, ermB*^1^	NT
Farm-B	Pig/7	*E. faecalis*	7	CLO^7^-TET^7^-ERY^7^-CIP^6^-GEN^7^-STR^7^	12–16	*fexA*^7^*, optrA*^7^*, tetM*^7^*, ermB*^7^*, aac6'-aph2''*^7^*, ant6′*^7^	ST330
	Pig/2	*E. faecalis*	2	TET^2^-ERY^2^-CIP^2^-STR^2^	NT	*ermB*^2^*, ant6′*^2^	NT
	2^nd^ Pig-farmer	*E. faecalis*	2	CLO^2^-TET^2^-ERY^2^-CIP^2^-GEN^2^-STR^2^	8, 10	*fexA*^2^*, optrA*^2^*, tetM*^2^*, ermB*^2^*, aac6'-aph2''*^2^*, ant6′*^2^	ST330
	1^st^ Pig-farmer	*E. faecalis*	1	CLO^1^-TET^1^-ERY^1^-CIP^1^-GEN^1^-STR^1^	NT	*tetM*^1^*, ermB*^1^*, aac6'-aph2''*^1^*, ant6′*^1^	NT
	3^rd^ Pig-farmer	*E. faecium*	1	PEN^1^-TET^1^-ERY^1^-CIP^1^	NT	*tetM*^1^*, ermB*^1^	NT
Farm-C	Pig/1	*E. faecalis*	1	TET^1^-ERY^1^-CIP^1^-STR^1^	NT	*tetM*^1^*, ermA*^1^*, ant6′*^1^	NT
	Pig/1	*E. faecalis*	1	PEN^1^-TET^1^-CIP^1^-STR^1^	NT	*tetM*^1^*, ant6′*^1^	NT
	Pig/1	*E. faecalis*	1	PEN^1^-TET^1^-CIP^1^	NT	*tetM*^1^	NT
	Pig/1	*E. faecium*	1	TET^1^-ERY^1^–CIP^1^-GEN^1^-STR^1^	NT	*tetM*^1^*, ermB*^1^*, aac6'-aph2''*^1^*, ant6′*^1^*,*	NT
	Pig/1	*E. faecium*	1	PEN^1^-TET^1^-CIP^1^	NT	*tetM*^1^	NT
	Pig/1	*E. gallinarum*	1	PEN^1^ -TET^1^-STR^1^	NT	*tetM*^1^*, ant6′*^1^	NT
	Pig/1	*E. gallinarum*	1	TET^1^-ERY^1^-CIP^1^-STR^1^	NT	*tetM*^1^*, ermB*^1^*, ant6*^1^*',*	NT
	Pig/1	*E. hirae*	1	PEN^1^-TET^1^-ERY^1^-CIP^1^	NT	*tetM*^1^*, ermA*^1^*, ermB*^1^	NT
	2^nd^ Pig-farmer	*E. faecium*	1	PEN^1^-TET^1^	NT	*tetM*^1^	NT
Farm-D	Pig/4	*E. faecalis*	6	CLO^6^-TET^6^-ERY^6^-CIP^6^-GEN^6^-STR^6^	2–3	*fexA*^6^*, catA*^6^*, cfrD*^6^*, optrA*^6^*, ermB*^6^*, tetM*^6^*, aac6'-aph2''*^6^*, ant6′*^6^	ST59
	Pig/1 Pig/1 Pig/1	*E. faecalis* *E. faecalis* *E. faecalis*	2 1 1	CLO^2^-TET^2^-ERY^2^-CIP^2^-GEN^2^-STR^2^ CLO^1^-TET^1^-ERY^1^-GEN^1^-STR^1^ CLO^1^-TET^1^-ERY^1^-GEN^1^-STR^1^	NT NT 12	*catA*^2^, *tetM*^2^*, ermB*^2^*, aac6'-aph2''*^2^*, ant6′*^*2*^ *catA*^1^*, tetM*^1^*, ermB*^1^*, aac6'-aph2''*^1^*, ant6′*^1^ *fexA*^1^*, optrA*^1^*, tetM*^1^*, ermB*^1^* aac6'-aph2''*^1^*, ant6′*^1^	NT NT ST330
	Pig/3	*E. faecalis*	4	TET^4^-ERY^4^-GEN^4^-STR^4^	NT	*tetM*^4^*, ermB*^4^*, aac6'-aph2''*^4^	NT
	Pig/1	*E. faecalis*	2	CLO^2^-TET^2^-ERY^2^	NT	*tetK*^2^*, tetM*^2^*, ermB*^2^	NT
	Pig/1	*E. faecalis*	2	CLO^2^-TET^2^-ERY^2^-STR^2^	NT	*catA*^*2*^*, ermB*^2^*, ant6′*^2^	NT
	Pig/1	*E. faecalis*	2	CLO^2^-TET^2^-ERY^2^- GEN^2^-STR^2^	8, 10	*fexA*^2^*, optrA*^2^*, aac6'-aph2''*^2^	ST474
	Pig/1	*E. faecium*	2	CLO^2^-TET^2^-ERY^2^-STR^2^	NT	*catA*^2^*, ermB*^2^, *ant6′*^2^	NT
	1^st^ Pig-farmer	*E. faecium*	2	PEN^2^-TET^2^-ERY^2^-STR^2^	NT	*ermB*^2^, *ant6′*^2^	NT
	2^nd^ Pig-farmer	*E. faecalis*	1	TET^1^-CIP^1^-STR^1^	NT	*tetM*^1^*, ant6′*^1^	NT

^a^In superscript is the number of isolates that present the specific phenotype/genotype of AMR; ST, sequence type; NT, not tested

^b^CLO, chloramphenicol; CIP, ciprofloxacin; ERY, erythromycin; GEN, gentamicin; LZD, linezolid; PEN, penicillin; STR, streptomycin; TET, tetracycline

^c^Linezolid MIC was tested in the isolates that carried linezolid resistance genes

Some pigs and pig-farmers were carriers of > 1 species and/ or same species with varied AMR phenotypes

None of the enterococci showed resistance to linezolid by disk diffusion; however, most of the chloramphenicol resistant isolates carried some acquired linezolid resistance genes (*optrA* and in some cases *cfrD*). Of special relevance was the detection of linezolid-resistance genes in enterococci of pigs: (a) in 33.3% of pigs tested (*E. faecalis* with *optrA* and/or *cfrD*; *E. casseliflavus* with *optrA* and *cfrD*); (b) in 10% of pig farmers (*E. faecalis* with *optrA*). The MLST of three *E. faecalis* isolates carrying the linezolid resistance genes from farms-A (pig) and B (pig and farmer) was performed and found to be of the genetic lineage ST330. However, the MLST of three of the linezolid resistant *E. faecalis* isolates from farm-D obtained from two pigs were ST330, ST474 and ST59 (Table 1). The isolates that carried linezolid resistance genes showed an MIC for linezolid in the range 8–16 µg/ml (Table 1).

### Nasal enterococcal carriage rate in healthy dogs and human household members

**Fig. 2:**
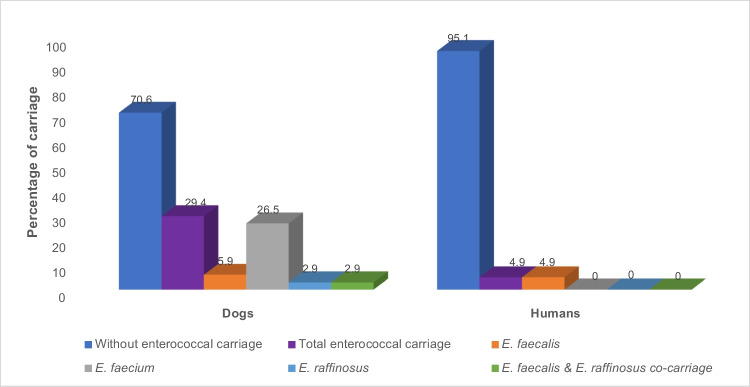
Nasal enterococcal carriage in healthy dogs and dog-owning human household members. The number of individuals sampled from dogs and human’s household members were 34 and 41 respectively from 27 households

Six out of the 27 households (22.2%) had nasal enterococci carriers**.** In total, 31 enterococci isolates (27 from dogs, 4 from human household members) were recovered. Of the dogs’ isolates, 18, 7 and 2 were *E. faecium*, *E. faecalis*, and *E. raffinosus*, respectively. However, all the isolates of humans were *E. faecalis* (*n* = 4) (Fig. 2). The nasal carriage rate of enterococci among healthy dogs and dog-owning humans were 29.4% and 4.9%, respectively.

### Antimicrobial resistomes of *Enterococcus* sp. isolates from healthy dogs and dog-owning human household members

In one of the 6 households with enterococci carriage (household ID number 10), both humans and dogs were *E. faecalis* carriers and the isolates presented a similar AMR phenotype and genotype (*tetM* positive) (Table 2). Moreover, in another household (household ID number 18), enterococci were detected in both humans and dogs, but belonged to different species (*E. faecalis* in humans and *E. faecium* in dogs). One of the dogs analysed in this study (household ID number 18) carried linezolid-resistant *E. faecalis* isolates that contained the *fexA*, *optrA*, *tetL*, *tetM*, *ermA*, *ermB*, *str*, *aac6'-aph2''*, and *ant6'* genes, and were typed as ST585.

**Table 2:** Intra-sample and intra-species variation of resistomes of *Enterococcus* sp. isolates from healthy dogs’ household

Host/ID no	Household ID no/ population	Species	No of isolates	AMR phenotypes^a,b^	AMR genes detected^a^	LZD MIC (μg/ml)^c^	Sequence type
Dog/ 28	11/ 2 humans, 2 dogs	*E. faecium*	1	PEN^1^-TET^1^-CIP^1^	*tetM*^1^	NT	NT
Dog/ 29	11/ 2 humans, 2 dogs	*E. faecium*	3	PEN^3^-ERY^3^-CIP^3^	ND^3^	NT	NT
Human/ 23	10/ 2 humans, 2 dogs	*E. faecalis* *E. faecalis*	1 1	PEN^1^-TET^1^ PEN^1^-CIP^1^	*tetM*^1^ ND	NT NT	NT NT
Dog/ 25	10/ 2 humans, 2 dogs	*E. faecalis* *E. faecalis* *E. raffinosus*	1 2 2	PEN^1^-TET^1^-CIP^1^ -GEN^1^ PEN^2^-TET^2^-CIP^2^ PEN^2^	*aac6'-aph2''*^1^*, tetM*^1^ *tetM*^2^ ND^2^	NT NT NT	NT NT NT
Human/ 47	18/ 2 humans, 2 dogs	*E. faecalis*	2	TET^2^-ERY^2^-CIP^2^-GEN^2^	*aac6'-aph2''*^*2*^*, ant6′*^2^*, tetM*^2^	NT	NT
Dog/ 48	18/ 2 humans, 2 dogs	*E. faecium* *E. faecium* *E. faecium* *E. faecium*	1 1 1 1	TET^1^ CHLCLO^1^-TET^1^ TET^1^-CIP^1^ TET^1^-CIP^1^	*tetM*^1^ *catA*^1^*, tetM*^1^ *tetM*^1^ *tetK*^1^*, tetL*^1^	NT NT NT NT	NT NT NT NT
Dog/ 49	18/ 2 humans, 2 dogs	*E. faecium* *E. faecium* *E. faecium* *E. faecium* *E. faecium*	1 1 1 1 1	TET^1^-CIP^1^ TET^1^ TET^1^-CIP^1^-STR^1^ TET^1^-CIP^1^ TET^1^	*tetM*^1^ *tetM*^1^ *tetM*^1^ *tetM*^1^ *tetM*^1^	NT NT NT NT NT	NT NT NT NT NT
Dog/ 53	19/ 2 humans, 2 dogs	*E. faecium* *E. faecium* *E. faecium*	1 1 1	PEN^1^-LZD^1^-ERY^1^-TET^1^-STR^1^ PEN^1^-TET^1^- ERY^1^-STR^1^ PEN^1^-TET^1^- ERY^1^-STR^1^	*tetM*^1^*, ermB*^1^*, ant6′*^1^ *ant6*^1^*', tetM*^1^ *ant6′*^1^*, tetM*^1^	NT NT NT	NT NT NT
Dog/ 56	20/ 2 humans, 2 dogs	*E. faecalis*	4	CHL-CLO^4^-LZD^4^-TET^4^-ERY^4^-CIP^4^-STR-GEN^4^	*fexA*^4^*, optrA*^4^*, tetL*^*4*^*, tetM*^4^*, ermA*^4^*, ermB*^*4*^*, str*^4^*, aac6'-aph2''*^4^*, ant6′*^4^	10–12	ST585
Dog/ 73	27/ 3 humans, 1 dog	*E. faecium*	2	Suceptible^2^	NT	NT	NT

^a^In superscript is the number of isolates that present the specific phenotype/genotype of AMR; ST, sequence type; NT, not tested; *ND*, not detected

^b^CHL, chloramphenicol; CIP, ciprofloxacin; ERY, erythromycin; GEN, gentamicin; LZD, linezolid; PEN, penicillin; STR, streptomycin; TET, tetracycline

^c^Linezolid MIC was tested in the isolates that carried linezolid resistance genes

Some pigs dogs were carriers of > 1 species and/or same species with varied AMR phenotypes

### Antimicrobial resistomes in the *Enterococcus* isolates from white stork nestlings

More than 70% of the 144 *Enterococcus* sp. from nasal and tracheal samples of stork origin studied were susceptible to all antibiotics tested (Table 3). However, 13.2% of enterococci showed tetracycline resistance, all of them of the species *E. faecalis* and *E. faecium*, and they carried the *tetM* and/or *tetK* genes (except in one *E. faecium* isolate); moreover, between 4 and 5% of enterococci showed erythromycin resistance (with *ermB *and* ermA* genes) and high-level aminoglycoside resistance (with *aac6'-aph2''* or *str* genes) (Table 3). In addition, and for the first time in this animal species, an *E. faecium* isolate was found carrying an acquired linezolid resistance gene (*poxtA*), in addition to *fexB* gene (associated to chloramphenicol resistance); this strain belonged to the lineage ST1736 and presented an MIC for linezolid of 8 µg/ml. None of the *E. casseliflavus*, *E. hirae*, *E. durans*, and *E. gallinarum* isolates of stork origin showed resistance to the antibiotics tested (Table 3).

**Table 3:** Antimicrobial resistomes of enterococci from nestlings based on foraging habit of parent storks

Sample type	No. of storks	*Enterococcus* species	AMR phenotype^a^	AMR genes detected	LZD MIC (μg/ml)^b^	Foraging habitat	Sequence type
Tracheal	30	*E. faecalis*	Susceptible	NT^c^	NT	Landfill	NT
Tracheal	6	*E. faecalis*	Susceptible	NT	NT	Natural	NT
Tracheal	2	*E. faecalis*	TET	*tetM*	NT	Landfill	NT
Tracheal	1	*E. faecalis*	TET	*tetM*	NT	Natural	NT
Tracheal	1	*E. faecalis*	TET-ERY	*tetK, tetM, ermB*	NT	Landfill	NT
Tracheal	1	*E. faecalis*	TET-ERY-STR	*tetM, ermB, str*	NT	Landfill	NT
Tracheal	1	*E. faecalis*	TET-ERY	*tetM, ermA, ermB*	NT	Landfill	NT
Tracheal	1	*E. faecalis*	ERY-GEN	*ermB, aac6'-aph2''*	NT	Landfill	NT
Tracheal	1	*E. faecalis*	TET-STR	*tetK, str*	NT	Landfill	NT
Tracheal	1	*E. hirae*	Susceptible	NT	NT	Natural	NT
Tracheal	1	*E. gallinarum*	Susceptible	NT	NT	Landfill	NT
Tracheal	1	*E. cecorum*	Susceptible	NT	NT	Natural	NT
Tracheal	6	*E. cecorum*	Susceptible	NT	NT	Landfill	NT
Tracheal	3	*E. faecium*	Susceptible	NT	NT	Natural	NT
Tracheal	2	*E. faecium*	Susceptible	NT	NT	Landfill	NT
Tracheal	1	*E. faecium*	PEN-CLO	*fexB, poxtA*	8	Landfill	ST1736
Tracheal	1	*E. faecium*	TET-CIP	*tetM*	NT	Landfill	NT
Tracheal	1	*E. faecium*	TET-CIP	ND	NT	Landfill	NT
Tracheal	3	*E. faecium*	CIP	ND	NT	Landfill	NT
Nasal	3	*E. faecium*	CIP	ND	NT	Landfill	NT
Nasal	7	*E. faecium*	Susceptible	NT	NT	Landfill	NT
Nasal	1	*E. faecium*	TET	*tetM*	NT	Landfill	NT
Nasal	1	*E. faecium*	TET	*tetM*	NT	Landfill	NT
Nasal	1	*E. canis*	Susceptible	NT	NT	Natural	NT
Nasal	3	*E. faecalis*	TET	*tetM*	NT	Landfill	NT
Nasal	3	*E. faecalis*	TET-STR	*tetK, tetM, str*	NT	Landfill	NT
Nasal	1	*E. faecalis*	TET-ERY	*tetK, tetM, ermB*	NT	Landfill	NT
Nasal	1	*E. faecalis*	TET-ERY	*tetK, tetM, ermB*	NT	Landfill	NT
Nasal	23	*E. faecalis*	Susceptible	NT	NT	Landfill	NT
Nasal	3	*E. faecalis*	Susceptible	NT	NT	Natural	NT
Nasal	4	*E. casseliflavus*	Susceptible	NT	NT	Natural	NT
Nasal	1	*E. gallinarum*	Susceptible	NT	NT	Landfill	NT
Nasal	1	*E. durans*	Susceptible	NT	NT	Natural	NT

^a^CLO, chloramphenicol, CIP, ciprofloxacin; ERY, erythromycin; GEN, gentamicin; LZD, linezolid; PEN, penicillin; STR, streptomycin; TET, tetracycline

^b^Linezolid MIC was tested in the isolates that carried linezolid resistance genes

^c^NT, not tested

### Comparison of AMR phenotype frequencies among *E. faecalis and E. faecium*

To compare the AMR frequencies of distinct *E. faecalis* and *E. faecium* isolates from dogs, pigs and storks’ nasal samples, individual chi-squared tests against every antimicrobial agent were computed. The prevalence of tetracycline, erythromycin, chloramphenicol, gentamicin, linezolid, and streptomycin resistances was significantly higher among *E. faecalis* of pigs than in the other two groups (*p* < 0.0001) (Table 4). All the enterococci carrying linezolid resistance genes were phenotypically susceptible by disc diffusion tests; however, upon LZD Etest for their MIC, all were found resistant (range: 8 to 16 μg/ml) except six isolates (all of the same animal) with an MIC of 2–3 μg/ml (Tables 1, 2 and 3).

**Table 4:** Comparison of the AMR phenotype frequencies among distinct *E. faecalis* and *E. faecium* isolates from dogs, pigs and white stork nestlings

Antimicrobial agent	*E. faecalis*			*χ*^2^	*p* value	*E. faecium*			*χ*^2^	*p* value
	No. (%) of resistant isolates from dogs (*n* = 3)	No. (%) of resistant isolates from pigs (*n* = 30)	No. (%) of resistant isolates from storks’ nasal samples (*n* = 34)			No. (%) of resistant isolates from dogs (*n* = 15)	No. (%) of resistant isolates from pigs (*n* = 4)	No. (%) of resistant isolates from storks’ nasal samples (*n* = 25)		
Penicillin	2 (66.7)	0 (0.0)	0 (0.0)	43.979	< 0.0001*	3 (20.0)	0 (0.0)	0 (0.0)	6.224	0.0445*
Tetracycline	3 (100.0)	30 (100.0)	8 (23.5)	41.238	< 0.0001*	14 (93.3)	4 (100.0)	2 (8.0)	32.814	< 0.0001*
Erythromycin	1 (33.3)	30 (100.0)	2 (5.9)	56.802	< 0.0001*	3 (20.0)	3 (75.0)	0 (0.0)	17.253	< 0.0001*
Ciprofloxacin	3 (100.0)	28 (93.3)	0 (0.0)	59.492	< 0.0001*	7 (46.7)	2 (50.0)	3 (12.0)	6.826	0.03294*
Gentamicin	2 (66.7)	24 (80.0)	0 (0.0)	43.979	< 0.0001*	0 (0.0)	1 (25.0)	0 (0.0)	10.233	0.00599*
Chloramphenicol	1 (33.3)	28 (93.3)	0 (0.0)	56.68	< 0.0001*	0 (0.0)	0 (0.0)	0 (0.0)	NA	NA
Linezolid	1 (33.3)	20 (66.7)	0 (0.0)	32.922	< 0.0001*	0 (0.0)	0 (0.0)	0 (0.0)	NA	NA
Streptomycin	1 (33.3)	26 (86.7)	3 (8.8)	39.222	< 0.0001*	4 (26.7)	3 (75.0)	0 (0.0)	16.467	0.00026^a^
Vancomycin	0 (0.0)	0 (0.0)	0 (0.0)	NA	NA	0 (0.0)	0 (0.0)	0 (0.0)	NA	NA

^a^Significant association determined by two tailed chi squared test at 95% Confidence interval (CI)

Percentages were based on the total *E. faecalis* and *E. faecium* isolates with distinct AMR profile

Concerning *E. faecium* isolates, penicillin resistance was significantly present among isolates of dogs than in the other two groups (*p* < 0.05) (Table 4). However, gentamicin, erythromycin, ciprofloxacin, and streptomycin resistances were significantly higher among *E. faecium* of pigs than in the other two groups (Table 4). In all cases, storks’ nasal *E. faecalis* and *E. faecium* isolates had the least AMR rates compared to the dogs’ and pigs’ isolates.

**Fig. 3:**
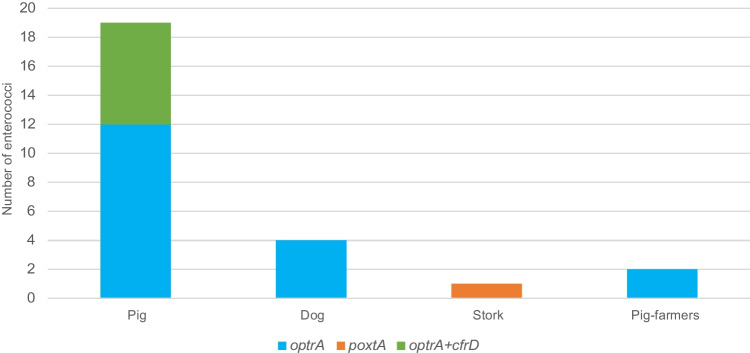
Distribution of acquired linezolid resistance genes among the four hosts

Among the chloramphenicol-resistant enterococci, notably many also harbouring linezolid resistance genes (*optrA*, *poxtA*, and *cfrD*) were detected in 16 pigs (33.3%), 1 dog (2.9%), 1 stork (1.1%) and 1 pig-farmer (10.0%) (Fig. 3).

### Risk factors associated with nasal enterococcal carriage and MDR phenotypes

After bivariate logistic analysis, nasal carriage (OR = 6.33, 95% CI: 2.29–17.42, *p* = 0.004) and occurrence rate of MDR phenotype (OR = 8.57, 95% CI: 2.99–24.56, *p* = 0.0001) were significantly associated with the species of animal (Supplementary Table S3). Although nasal enterococcal carriage in storks was double but not significantly different from that of dogs (OR = 2.4, 95% CI: 0.93–6.17, *p* = 0.069). Also, nasal enterococci carriage in humans was significantly associated with the species of animal contact (OR = 29.25, 95% CI: 4.36–196.07, *p* = 0.0005). Dog-owning households with > 1 dog & 1 human had relatively higher odds of nasal enterococci carriage than those with only 1 dog & 1 human; however, this was not statistically significant (OR = 3.75, 9% CI: 0.37–37.94, *p* = 0.268) (Supplementary Table S3).

## Discussion

We are not aware of any previous study that simultaneously investigated the nasal enterococci communities of food-producing animals, pets and wild animals. Perhaps because they are frequent intestinal commensals, most studies focus on the GI enterococci carriage [5].

Over the last 2 decades, our research group has detected ARGs to critical antimicrobials used as the last resort chemotherapy against enterococci infections in isolates from Spain, Portugal and Tunisia (especially, conferring resistance to vancomycin and linezolid) in wild boars (*vanA*-carrying *E. faecium*), wild rodents (*vanB2*-carrying *E. faecalis* and *vanA*-carrying *E. faecium*), wild birds (*vanA*- and *vanB2*-carrying *E. faecalis*), chickens (*vanA*-carrying *E. hirae*), pig environment air (*poxtA*- and *optrA*-carrying *E. faecium*) and clinical samples (*vanA/vanB E. faecalis* and *E. faecium*,* optrA*- and *cfr*D-carrying *E. faecalis*) [25–29]. However, none of them was on nasal samples.

In all the three animal hosts studied, the nasal carriage rate was high (especially in pigs and storks). The high nasal enterococci rate detected in our study highlights their frequent association of *Enterococcus* spp. with the respiratory tracts of the animals. Thus, it is essential to remark that enterococci are not only found at high rates in the GI tract but also in nasal samples, as demonstrated in this study. On the other hand, healthy dogs were relatively fewer carriers of enterococci, and this might be due to host adaption differences to respiratory epithelia.

There is growing evidence that the use of chloramphenicol chemotherapy in animal husbandry can select for enterococci harbouring *optrA* and *poxtA* genes which confer resistance to the critically important antibiotic linezolid, posing a risk to human health via the food chain and contact with livestock.

In this study, the majority (over 90%) of the enterococci carrying oxazolidinone resistance genes belonged to *E. faecalis* or *E. faecium*, which are the predominant *Enterococcus* species in humans and animals (including pets and pigs), but also belong to the most important Gram-positive nosocomial pathogens worldwide [4]. We found a significantly higher frequency of LRE in pigs than what was reported in comparable studies on faecal samples from pigs in Switzerland (5%), Belgium (11%) and Italy (21%) [12, 13, 30]. Notably, comparative data are still scarce and variations between countries for which data are available should be interpreted with caution due to the differences in study designs, sample types and testing methodologies. Nevertheless, the present study indicates that the occurrence of chloramphenicol-resistant enterococci among our pigs are high. Worryingly, the use of antibiotics in pig farming in recent years has been very high in Spain, highlighting the need to raise awareness within the agricultural sector to mitigate the emergence and spread of linezolid-resistant enterococci in the future. Moreover, most of the enterococci in this study were associated with the presence of tetracycline resistance genes. Tetracycline is the most frequent veterinary antibiotic used for treating many swine bacterial diseases and is likely to promote the spread and persistence of LRE in pigs [31, 32]*.* The *optrA*-carrying-*E. faecalis*-ST330, -ST474 and -ST59 circulating in 3 of the 4 studied farms have been previously reported in human and many animal hosts such as macaques, pigs, chickens, poultry meat, and vultures [13, 30, 33–40]. These *optrA*-positive lineages appear to be non-host specific.

The detection of LRE in pig farmers and a dog indicate potential risk of transmission to other humans and animals outside the pigs-farm environment and dog-owning households, respectively. These put together with the several *optrA-*positive *E. faecalis* isolates reported in dogs fed with raw meat/vegetables in China [41] underscore relevance of the ‘One-Health’ approach for investigating LRE, as they can be shared by animals, humans and environnment. However, the direction of transfer is often difficult to prove, especially as none of the humans in contact with the dogs were carriers of LRE. Currently, the knowledge of the LRE prevalence in companion animals is limited and therefore a joint approach to monitor the emergence and dissemination of resistance mechanisms of public health importance are needed. The MDR *E. faecalis*-ST585 isolate detected in a dog in our study was similar to previously reported LR-*E. faecalis* isolates from Spanish hospitals [42]. Moreover, this is the first description of ST585 carrying the *optrA* gene in dogs from Spain.

Plasmid-encoded *optrA* and *poxtA* in *E. durans* and *E. hirae* were previously reported in pigs in Switzerland, as well as *poxtA-*carrying *E. hirae* from China and Italy [13, 29, 31, 43] and *optrA-*carrying *E. gallinarum* from a fattening pig in Belgium [12] were recently identified. Also, a *cfrD*-carrying *E. casseliflavus* strain has recently been reported from pigs’ manure in Italy [44] and *optrA*/*cfr*-carrying *E. casseliflavus* from a faecal swab of a pig in China [45]. To the best of our knowledge, the detection of *E. casseliflavus* carrying *optrA* and *cfrD* in a pig in our study is the first report. These put together suggest that pigs could be potential reservoirs for the dissemination and persistence of *E. casseliflavus* carrying various linezolid transferable resistance genes. As *E. casseliflavus* only occasionally causes opportunistic infections in humans [4], the presence of linezolid resistance genes in this species from pigs may not pose a direct threat to human health but could play an important role in transferring this resistance mechanism. It is worth mentioning that none of the isolates had mutation in their 23S rDNA.

Concerning the stork’s *E. faecium*-ST1736 carrying *poxtA* in our study, migratory birds may be an important link in the spread of LRE. This isolate was obtained from a nestling that was feed food foraged by its parents in the landfills; so, the exposure could be from human household residues, landfill discarded wastewater treatment plant slurry or animal remains. This is the first time that LR-*E. faecium* ST1736 has been reported in storks. The detection of linezolid resistance genes is highly relevant since these genes could be in plasmids and be transmitted to clinical settings, production animals or the environment.

It is of interest to remark that all the linezolid resistant enterococci were recovered in the ChromAgar LIN agar plates in which isolates were grown as green colonies. Nevertheless, linezolid susceptible isolates were also recovered in this media, as also indicated by other authors [24].

In storks, a vast majority of the *Enterococcus* species were susceptible to all the antibiotics tested. This difference may reflect the level of selection pressure, particularly due to the extensive use of antibiotics in pig-farming as compared to antibiotic chemotherapy in dogs and humans [46]. Although vancomycin-resistant enterococci (VRE) are considered high-priority pathogens of great public health concern resistance [47], none of the isolates carried the *vanA* and *vanB* genes. Notably, the detected AMR genes in E. faecium or faecalis isolates from storks were all from landfill-associated colonies (except one). Most likely, the individuals were fed landfill foraged food by their parents.

Both acquired and intrinsic resistance properties drastically reduce the options for antimicrobial therapy. Bearing this in mind, the performance of antimicrobial susceptibility tests prior to the start of antimicrobial therapy is of particular significance to guide the application of antimicrobial agents in pigs-farming and canine medicine.

## Conclusion

*Enterococcus casseliflavus* carrying *optrA* and *cfrD* is described for the first time in pigs. This put together with the occurrence of *cfrD* and *optrA* in *E. faecalis*-ST585 and -ST330, *poxtA* in *E. faecium*-ST1736 in healthy dogs, pigs and storks emphasizes the potential risk to human health through the dissemination of LRE﻿﻿ in the food chain and companion and wild animals. The *poxtA* gene is described for the first time in an *E. faecium *from a migratory bird that could facilitate its spread to other ecosystems. Foraging of the parents of this stork in landfills could explain the acquisition of this multidrug-resistant strain. The presence of linezolid resistance genes with potential of horizontal transfer may go unnoticed by disc diffusion phenotypic tests, unless they are detected by MIC determination. Nevertheless, the selective inclusion of chloramphenicol resistance phenotype as marker to screen for linezolid resistance genes in enterococci may be advantageous for their detection at a molecular level. Our results showed that the nasal cavity of pigs, dogs and the trachea of storks may represent an important source of LRE, possibly contributing to animal-to-human transmission and transmission to the environment or food by these colonized animals and people.

## Supplementary Information

Below is the link to the electronic supplementary material.

Supplementary file1 (DOCX 19 KB)

Supplementary file2 (DOCX 31 KB)

Supplementary file3 (DOCX 15 KB)

## Data Availability

The data generated from this study can be available on request through the corresponding author (Carmen Torres).
