# Supplementary material for: Nasotracheal enterococcal carriage and resistomes: detection of optrA-, poxtA- and cfrD-carrying strains in migratory birds, livestock, pets, and in-contact humans in Spain
Source: Eur J Clin Microbiol Infect Dis. 2023 Mar 9;42(5):569–81. doi: 10.1007/s10096-023-04579-9 (PMC10105672; doi:10.1007/s10096-023-04579-9)
Supplement: Supplementary file 1 — Supplementary file1 (DOCX 19 KB) [file 10096_2023_4579_MOESM1_ESM.docx]

**Table S1**. Enterococci strains from all healthy individuals and those with distinct AMR phenotypes

| Origin | Total isolates | N^o^ of *E. faecalis* | N^o^ of *E. faecium* | N^o^ of *E. casselifavus* | N^o^ of *E. gallinarum* | N^o^ of *E. durans* | N^o^ of *E. hirae* | N^o^ of *E. canis* | N^o^ of *E. cecorum* | N^o^ of *E. raffinosus* | Distinct *E. faecalis* isolates^a^ | Distinct *E. faecium* isolates^a^ |
| --- | --- | --- | --- | --- | --- | --- | --- | --- | --- | --- | --- | --- |
| Dog households  Dogs  Humans | 31  27  4 | 11  7  4 | 18  18  0 | 0  0  0 | 0  0  0 | 0  0  0 | 0  0  0 | 0  0  0 | 0  0  0 | 2  2  0 | 6  3  3 | 15  15  0 |
| Pig farms  Pigs  Pigs-personnel | 51  43  8 | 38  34  4 | 8  4  4 | 1  1  0 | 2  2  0 | 0  0  0 | 2  2  0 | 0  0  0 | 0  0  0 | 0  0  0 | 32  30  2 | 7  4  3 |
| Storks  Tracheal  Nasal | 144  74  70 | 78  44  34 | 47  19  28 | 5  0  5 | 2  1  1 | 2  0  2 | 1  1  0 | 1  1  0 | 8  8  0 | 0  0  0 | 78  44  34 | 44  19  25 |

^a^Distinct isolates are those from different samples or those of the same sample but of different AMR phenotype
