# Supplementary material for: Nasotracheal enterococcal carriage and resistomes: detection of optrA-, poxtA- and cfrD-carrying strains in migratory birds, livestock, pets, and in-contact humans in Spain
Source: Eur J Clin Microbiol Infect Dis. 2023 Mar 9;42(5):569–81. doi: 10.1007/s10096-023-04579-9 (PMC10105672; doi:10.1007/s10096-023-04579-9)
Supplement: Supplementary file 2 — Supplementary file2 (DOCX 31 KB) [file 10096_2023_4579_MOESM2_ESM.docx]

| **Gene** | **Primers’ oligonucleotide (5’**⇒**3’)** | **Amplicon size** | **Reference** |
| --- | --- | --- | --- |
| **Antimicrobial resistance genes** | | | |
| *ermA* | F: TCTAAAAAGCATGTAAAAGAA | 645 bp | [1] |
|  | R: CTTCGATAGTTTATTAATATTAG |  |  |
| *ermB* | F: GAAAAGTACTCAACCAAATA | 639 bp | [1] |
|  | R: AGTAACGGTACTTAAATTGTTTA |  |  |
| *ermC* | F: TCAAAACATAATATAGATAAA | 642 bp | [1] |
|  | R: GCTAATATTGTTTAAATCGTCAAT |  |  |
| *ermT* | F: CCGCCATTGAAATAGATCCT | 200 bp | [2] |
|  | R: TTCTGTAGCTGTGCTTTCAAAAA |  |  |
| *aac6’-aph2’’* | F: CCAAGAGCAATAAGGGCATA | 220 bp | [3] |
|  | R: CACTATCATAACCACTACCG |  |  |
| *ant*6’ | F: ACTGGCTTAATCAATTTGGG | 597 bp | [4] |
|  | R: TTATTGATAATTTTGGTT |  |  |
| *str* | F: TATTGCTCTCGAGGGTTC | 646 bp | [5] |
|  | R: CTTTCTATATCCATTCATCTC |  |  |
| *tetL* | F: CATTTGGTCTTATTGGATCG | 456 bp | [6] |
|  | R: ATTACACTTCCGATTTCGG |  |  |
| *tetM* | F: GTTAAATAGTGTTCTTGGAG | 576 bp | [6] |
|  | R: CTAAGATATGGCTCTAACAA |  |  |
| *tetK* | F: TTAGGTGAAGGGTTAGGTCC | 697 bp | [6] |
|  | R: GCAAACTCATTCCAGAAGCA |  |  |
| *cat_pC221_* | F: ATTTATGCAATTATGGAAGTTG | 434 bp | [5] |
|  | R: TGAAGCATGGTAACCATCAC |  |  |
| *cat_pC223_* | F: GAATCAAATGCTAGTTTTAACTC | 283 bp | [5] |
|  | R: ACATGGTAACCATCACATAC |  |  |
| *cat_pC194_* | F: CGACTTTTAGTATAACCACAGA | 570 bp | [5] |
|  | R: GCCAGTCATTAGGCCTAT |  |  |
| *catA* | F: GGATATGAAATTTATCCCTC | 505 bp | [6] |
|  | R: CAATCATCTACCCTATGAAT |  |  |
| *fexA* | F: GTACTTGTAGGTGCAATTACGGCTGA | 1272 bp | [6] |
|  | R: CGCATCTGAGTAGGACATAGCGTC |  |  |
| *fexB* | F: TTCCCACTATTGGTGAAAGGAT | 816 bp | [7] |
|  | R: GCAATTCCCTTTTATGGACGTT |  |  |
| *cfr* | F: TGAAGTATAAAGCAGGTTGGGAGTCA | 746 bp | [8] |
|  | R: ACCATATAATTGACCACAAGCAGC |  |  |
| *cfrB* | F: TGAGCATATACGAGTAACCTCAAGA | 293 bp | [9] |
|  | R: CGCAAGCAGCGTCTATATCA |  |  |
| *cfrD* | F: AGAAGTCGCAACAAGTGAGGA | 595 bp | [10] |
|  | R: GCAACTGCATGAGTCAAAGAA |  |  |
| *optrA* | F: AGGTGGTCAGCGAACTAA | 1395 bp | [11] |
|  | R: ATCAACTGTTCCCATTCA |  |  |
| *poxtA* | F: TCAATGCAGAGCAGGAAGCA | 791 bp | [10] |
|  | R: GGTGGATTTACCGACACCGT |  |  |
| ***E. faecalis* housekeeping alleles for MLST** | | | |
| *aroE* | F: TGGAAAACTTTACGGAGACAGC | 459 pb | [12] |
|  | R: GTCCTGTCCATTGTTCAAAAGC |  |  |
| *gdh* | F: GGCGCACTAAAAGATATGGT | 530 pb | [12] |
|  | R: CCAAGATTGGGCAACTTCGTCCCA |  |  |
| *gki* | F: GATTTTGTGGGAATTGGTATGG | 438 pb | [12] |
|  | R: ACCATTAAAGCAAAATGATCGC |  |  |
| *gyd* | F: CAAACTGCTTAGCTCCAAGGC | 395 bp | [12] |
|  | R: CATTTCGTTGTCATACCAAGC |  |  |
| *pstS* | F: CGGAACAGGACTTTCGC | 583 bp | [12] |
|  | R: ATTTACATCACGTTCTACTTGC |  |  |
| *xpt* | F: AAAATGATGGCCGTGTATTAGG | 456 bp | [12] |
|  | R: AACGTCACCGTTCCTTCACTTA |  |  |
| *yqiL* | F: CAGCTTAAGTCAAGTAAGTGCCG | 436 bp | [12] |
|  | R: GAATATCCCTTCTGCTTGTGCT |  |  |
| ***E. faecium* housekeeping alleles for MLST** | | | |
| *adK* | F: TATGAACCTCATTTTAATGGG | 437 bp | [13] |
|  | R: GTTGACTGCCAAACGATTTT |  |  |
| *atpA* | F: CGGTTCATACGGAATGGCACA | 556 bp | [13] |
|  | R: AAGTTCACGATAAGCCACGG |  |  |
| *ddl* | F: GAGACATTGAATATGCCTTAT | 465 bp | [13] |
|  | R: AAAAAGAAATCGCACCG |  |  |
| *gdh* | F: GGCGCACTAAAAGATATGGT | 530 bp | [13] |
|  | R: CCAAGATTGGGCAACTTCGTCCCA |  |  |
| *gyd* | F: CAAACTGCTTAGCTCCAAGGC | 395 bp | [13] |
|  | R: CATTTCGTTGTCATACCAAGC |  |  |
| *pstS* | F: TTGAGCCAAGTCGAAGCTGGA | 583 bp | [13] |
|  | R: CGTGATCACGTTCTACTTCC |  |  |
| *purK* | F: GCAGATTGGCACATTGAAAGT | 492 bp | [13] |
|  | R: TACATAAATCCCGCCTGTTTY |  |  |

1. Sutcliffe J, Grebe T, Tait-Kamradt A, Wondrack L. Detection of erythromycin-resistant determinants by PCR. Antimicrob Agents Chemother. 1996 Nov;40(11):2562-6. doi: 10.1128/AAC.40.11.2562.
2. Gómez-Sanz E, Torres C, Lozano C, Fernández-Pérez R, Aspiroz C, Ruiz-Larrea F, Zarazaga M. Detection, molecular characterization, and clonal diversity of methicillin-resistant *Staphylococcus aureus* CC398 and CC97 in Spanish slaughter pigs of different age groups. Foodborne Pathog Dis. 2010 Oct;7(10):1269-77. doi: 10.1089/fpd.2010.0610.
3. van de Klundert J, Vliegenthart J. PCR detection of genes coding for aminoglycoside-modifying enzymes, in: Diagnostic Molecular Microbiology. Principles and Applications. 1993; pp. 547– 552. <https://doi.org/10.1023/A:1016601629518>
4. Clark NC, Olsvik O, Swenson JM, Spiegel CA, Tenover FC. Detection of a streptomycin/spectinomycin adenylyltransferase gene (aadA) in *Enterococcus faecalis*. Antimicrob Agents Chemother. 1999 Jan;43(1):157-60. doi: 10.1128/AAC.43.1.157.
5. Schnellmann C, Gerber V, Rossano A, Jaquier V, Panchaud Y, Doherr MG, Thomann A, Straub R, Perreten V. Presence of new *mecA* and *mph(C)* variants conferring antibiotic resistance in *Staphylococcus spp*. isolated from the skin of horses before and after clinic admission. J Clin Microbiol. 2006 Dec;44(12):4444-54. doi: 10.1128/JCM.00868-06.
6. Aarestrup FM, Agerso Y, Gerner-Smidt P, Madsen M, Jensen LB. Comparison of antimicrobial resistance phenotypes and resistance genes in *Enterococcus faecalis* and *Enterococcus faecium* from humans in the community, broilers, and pigs in Denmark. Diagn Microbiol Infect Dis. 2000 Jun;37(2):127-37. doi: 10.1016/s0732-8893(00)00130-9.
7. Liu H, Wang Y, Wu C, Schwarz S, Shen Z, Jeon B, Ding S, Zhang Q, Shen J. A novel phenicol exporter gene, *fexB*, found in enterococci of animal origin. J Antimicrob Chemother. 2012 Feb;67(2):322-5. doi: 10.1093/jac/dkr481.
8. Kehrenberg C, Schwarz S. Distribution of florfenicol resistance genes *fexA* and *cfr* among chloramphenicol-resistant *Staphylococcus* isolates. Antimicrob Agents Chemother. 2006 Apr;50(4):1156-63. doi: 10.1128/AAC.50.4.1156-1163.2006.
9. Lee SM, Huh HJ, Song DJ, Shim HJ, Park KS, Kang CI, Ki CS, Lee NY. Resistance mechanisms of linezolid-nonsusceptible enterococci in Korea: low rate of 23S rRNA mutations in *Enterococcus faecium.* J Med Microbiol. 2017 Dec;66(12):1730-1735. doi: 10.1099/jmm.0.000637
10. Ruiz-Ripa L, Feßler AT, Hanke D, Eichhorn I, Azcona-Gutiérrez JM, Pérez-Moreno MO, Seral C, Aspiroz C, Alonso CA, Torres L, Alós JI, Schwarz S, Torres C. Mechanisms of Linezolid Resistance Among Enterococci of Clinical Origin in Spain-Detection of *optrA*- and *cfr*(D)-Carrying *E.faecalis*. Microorganisms. 2020 Jul 30;8(8):1155. doi: 10.3390/microorganisms8081155.
11. Wang Y, Lv Y, Cai J, Schwarz S, Cui L, Hu Z, Zhang R, Li J, Zhao Q, He T, Wang D, Wang Z, Shen Y, Li Y, Feßler AT, Wu C, Yu H, Deng X, Xia X, Shen J. A novel gene, *optrA,* that confers transferable resistance to oxazolidinones and phenicols and its presence in *Enterococcus faecalis* and *Enterococcus faecium* of human and animal origin. J Antimicrob Chemother. 2015 Aug;70(8):2182-90. doi: 10.1093/jac/dkv116.
12. Ruiz-Garbajosa P, Bonten MJ, Robinson DA, Top J, Nallapareddy SR, Torres C, Coque TM, Cantón R, Baquero F, Murray BE, del Campo R, Willems RJ. Multilocus sequence typing scheme for *Enterococcus faecalis* reveals hospital-adapted genetic complexes in a background of high rates of recombination. J Clin Microbiol. 2006 Jun;44(6):2220-8. doi: 10.1128/JCM.02596-05.
13. Homan WL, Tribe D, Poznanski S, Li M, Hogg G, Spalburg E, Van Embden JD, Willems RJ. Multilocus sequence typing scheme for *Enterococcus faecium*. J Clin Microbiol. 2002 Jun;40(6):1963-71. doi: 10.1128/JCM.40.6.1963-1971.2002
