# Supplementary material for: Nasotracheal enterococcal carriage and resistomes: detection of optrA-, poxtA- and cfrD-carrying strains in migratory birds, livestock, pets, and in-contact humans in Spain
Source: Eur J Clin Microbiol Infect Dis. 2023 Mar 9;42(5):569–81. doi: 10.1007/s10096-023-04579-9 (PMC10105672; doi:10.1007/s10096-023-04579-9)
Supplement: Supplementary file 3 — Supplementary file3 (DOCX 15 KB) [file 10096_2023_4579_MOESM3_ESM.docx]

**Table S3.** Risk factors associated with nasal enterococcal carriage and MDR phenotypes

| Variable | Enterococci presence (%) | Enterococci absence (%) | OR (95% CI) | *p* value |
| --- | --- | --- | --- | --- |
| Nasal carriage by animal type  Pigs (n=40)  Dogs (n=34)  Storks (n=52) | 29 (72.5)  10 (29.4)  36 (69.2) | 11 (27.5)  24 (70.6)  16 (30.7) | 1.17 (0.47-2.91)  0.19 (0.07-0.48)  Referent | 0.7329  0.0005^a^  Referent |
| MDR phenotype by animal type  Pigs (n=40)  Dogs (n=34)  Storks (n=52) | 29 (72.5)  8 (23.5)  3 (5.8) | 11 (27.5)  26 (76.5)  49 (94.2) | 43.06 (11.09-167.2)  5.02 (1.23-20.6)  Referent | 0.0001  0.025  Referent |
| Nasal carriage by animal contact  Pig farm personnel (n=10)  Pet ownership (n=41) | 6 (60.0)  2 (4.9) | 4 (40.0)  39 (95.1) | 29.25 (4.36-196.07)  Referent | 0.0005^a^  Referent |
| Nasal carriage by household density  >1 dogs &1 humans (n=17)  1 dog &1 human (n=10) | 5 (29.4)  1 (10.0) | 12 (70.6)  9 (90.0) | 3.75 (0.37-37.94)  Referent | 0.268  Referent |

^a^Significant association determined by bivariate regression at 95% Confidence interval (CI)

MDR: Multidrug resistance
